# Age is the main determinant of COVID-19 related in-hospital mortality with minimal impact of pre-existing comorbidities, a retrospective cohort study

**DOI:** 10.1186/s12877-021-02673-1

**Published:** 2022-03-05

**Authors:** M. T. H. M. Henkens, A. G. Raafs, J. A. J. Verdonschot, M. Linschoten, M. van Smeden, P. Wang, B. H. M. van der Hooft, R. Tieleman, M. L. F. Janssen, R. M. A. ter Bekke, M. R. Hazebroek, I. C. C. van der Horst, F. W. Asselbergs, F. J. H. Magdelijns, S. R. B. Heymans, on behalf of the CAPACITY-COVID collaborative consortium, A. K. Al-Ali, F. A. Al-Muhanna, N. Y. Y. Al-Windy, Y. A. Almubarak, A. N. Alnafie, M. Alshahrani, A. M. Alshehri, R. L. Anthonio, F. W. Asselbergs, A. Aujayeb, J. M. ten Berg, A. J. M. van Boxem, G. Captur, M. Caputo, N. Charlotte, P. Dark, J. De Sutter, C. E. Delsing, H. G. R. Dorman, J. T. Drost, M. E. Emans, J. B. Ferreira, L. Gabriel, W. H. van Gilst, B. E. Groenemeijer, H. E. Haerkens-Arends, P. van der Harst, B. Hedayat, D. J. van der Heijden, E. Hellou, R. S. Hermanides, J. F. Hermans-van Ast, M. W. J. van Hessen, S. R. B. Heymans, I. C. C. van der Horst, S. H. van Ierssel, L. S. Jewbali, M. T. Kearney, H. A. M. van Kesteren, B. L. J. H. Kietselaer, A. M. H. Koning, P. Y. Kopylov, A. F. M. Kuijper, J. M. Kwakkel-van Erp, M. M. J. M. van der Linden, M. Linschoten, G. C. M. Linssen, R. Macias Ruiz, F. J. H. Magdelijns, F. M. A. C. Martens, G. P. McCann, P. van der Meer, M. F. L. Meijs, P. Messiaen, P. S. Monraats, L. Montagna, A. Moriarty, A. Mosterd, P. R. Nierop, C. E. E. van Ofwegen-Hanekamp, Y. M. Pinto, H. Poorhosseini, S. Prasad, J. Redón, A. C. Reidinga, M. I. A. Ribeiro, D. P. Ripley, R. Salah, E. Saneei, M. Saxena, J. Schaap, D. A. A. M. Schellings, A. Schut, A. Shafiee, A. C. Shore, H. J. Siebelink, M. van Smeden, P. C. Smits, R. Pisters, E. Tessitore, R. G. Tieleman, P. Timmermans, R. A. Tio, F. V. Y. Tjong, C. A. den Uil, E. M. Van Craenenbroeck, H. P. A. A. van Veen, T. Veneman, D. O. Verschure, J. K. de Vries, R. M. A. van de Wal, D. J. van de Watering, I. C. D. Westendorp, P. H. M. Westendorp, C. Weytjens, E. Wierda, B. Williams, P. Woudstra, K. W. Wu, R. Zaal, A. G. Zaman, P. M. van der Zee

**Affiliations:** 1 Department of Cardiology, CARIM, Maastricht University Medical Center, P. Debyelaan 25, 6229 HX Maastricht, The Netherlands; 2 Netherlands Heart Institute (NLHI), Utrecht, The Netherlands; 3 Department of Clinical Genetics, CARIM, Maastricht University Medical Center, Maastricht, The Netherlands; 4 Department of Cardiology, Division of Heart and Lungs, University Medical Center Utrecht, Utrecht University, Utrecht, the Netherlands; 5 UMCU-Julius Center for Health Sciences and Primary Care, University Medical Center Utrecht, Utrecht University, Utrecht, the Netherlands; 6 Department of Internal Medicine, Maastricht University Medical Center, Maastricht, The Netherlands; 7 Department of Cardiology, Martini Hospital, Groningen, The Netherlands; 8 Department of Clinical Neurophysiology, Maastricht University Medical Center, Maastricht, The Netherlands; 9 Department of Intensive Care, Maastricht University Medical Center, Maastricht, The Netherlands; 10 Institute of Cardiovascular Science, Faculty of Population Health Sciences, University College London, London, UK; 11 Health Data Research UK and Institute of Health Informatics, University College London, London, UK; 12 Department of Cardiovascular Research, University of Leuven, Leuven, Belgium

**Keywords:** COVID-19, Mortality, Hospitalization, Netherlands, Mediation analysis

## Abstract

**Background:**

Age and comorbidities increase COVID-19 related in-hospital mortality risk, but the extent by which comorbidities mediate the impact of age remains unknown.

**Methods:**

In this multicenter retrospective cohort study with data from 45 Dutch hospitals, 4806 proven COVID-19 patients hospitalized in Dutch hospitals (between February and July 2020) from the CAPACITY-COVID registry were included (age 69[58–77]years, 64% men). The primary outcome was defined as a combination of in-hospital mortality or discharge with palliative care. Logistic regression analysis was performed to analyze the associations between sex, age, and comorbidities with the primary outcome. The effect of comorbidities on the relation of age with the primary outcome was evaluated using mediation analysis.

**Results:**

In-hospital COVID-19 related mortality occurred in 1108 (23%) patients, 836 (76%) were aged ≥70 years (70+). Both age 70+ and female sex were univariably associated with outcome (odds ratio [OR]4.68, 95%confidence interval [4.02–5.45], OR0.68[0.59–0.79], respectively;both *p*<  0.001). All comorbidities were univariably associated with outcome (*p*<0.001), and all but dyslipidemia remained significant after adjustment for age70+ and sex. The impact of comorbidities was attenuated after age-spline adjustment, only leaving female sex, diabetes mellitus (DM), chronic kidney disease (CKD), and chronic pulmonary obstructive disease (COPD) significantly associated (female OR0.65[0.55–0.75], DM OR1.47[1.26–1.72], CKD OR1.61[1.32–1.97], COPD OR1.30[1.07–1.59]). Pre-existing comorbidities in older patients negligibly (<6% in all comorbidities) mediated the association between higher age and outcome.

**Conclusions:**

Age is the main determinant of COVID-19 related in-hospital mortality, with negligible mediation effect of pre-existing comorbidities.

**Trial registration:**

CAPACITY-COVID (NCT04325412)

**Supplementary Information:**

The online version contains supplementary material available at 10.1186/s12877-021-02673-1.

## Background

COVID-19 is a pandemic infectious disease caused by the severe acute respiratory syndrome coronavirus 2 (SARS-CoV-19), which already accounts for over 5 million deaths worldwide [1]. COVID-19 related mortality is particularly high in the elderly [2–6]. Consequently, in the Netherlands, 90% of the deceased patients is 70 years or older [7].

Pre-existing comorbidities such as hypertension, diabetes mellitus (DM), dyslipidemia, chronic kidney disease (CKD), chronic obstructive pulmonary disease (COPD), and history of cardiac diseases are also associated with an increased risk of COVID-19 related (in-hospital) mortality [8–12]. However, these comorbidities are also known to be more prevalent in the elderly [8, 9, 13]. It remains unknown to which extent the higher in-hospital mortality rate is mediated by the higher prevalence of these comorbidities in the elderly.

The aim of this study was to explore to which extent existing comorbidities mediate the increased risk of COVID-19 related in-hospital mortality relative to age in a registry of 4806 Dutch COVID-19 patients.

## Methods

### Study design and population

CAPACITY-COVID (NCT04325412) is an international patient registry established to investigate the role of cardiovascular disease in the COVID-19 pandemic [14, 15]. The details of this registry have been outlined in detail previously [15]. In short, adult patients (≥18 years) with (highly suspected) COVID-19 admitted to one of the participating hospitals were included in this registry [14]. Forty-five Dutch hospitals contributed to the registry. For the current analysis, consecutive patients from all participating Dutch hospitals with proven COVID-19 infection based on at least one positive PCR for SARS-CoV-2 (92% of the included subjects) and/or a chest CT scan strongly suggestive for SARS-CoV-2 infection, hospitalized between January and July 2020 (first COVID-19 wave in the Netherlands), were included [14–16]. In-hospital mortality was defined as a combination of in-hospital mortality or discharge with palliative care. The study was performed according to the Helsinki declaration and local ethics approval was obtained in all participating hospitals. Consent was obtained by either opt-in (when required by the local Medical Research Ethics Committee of the participating center) or opt-out approaches, following local regulations.

### Statistical analyses

Variables are displayed as numbers (percentage), mean ± standard deviation (SD) or median and inter quartile ranges [IQR], as appropriate. Normality was assessed visually using Q-Q plots. Comparisons between groups were performed using chi-square tests for categorical data and for continuous variables by independent sample T-test or Mann Whitney-U test depending on normality of the distribution. Unadjusted binary logistic regression analysis was performed to analyze the associations between sex, age 60+, age 70+, a reported medical history of comorbidities (hypertension, DM, dyslipidemia, CKD, COPD, and a medical history of cardiac disease) and the comorbidity count (a sum of the presence of the before mentioned comorbidities categorized as 0 co-morbidities, 1–2 co-morbidities, and > 2 co-morbidities) with in-hospital mortality. Definitions of pre-existing comorbidities as specified in the case report form are provided in the Supplemental Methods (Additional file 1). Subsequently, adjusted binary logistic regression analysis – with adjustment for age 70+ and sex – was performed to determine the adjusted association between comorbidities and the comorbidity count with in-hospital mortality. Additionally, given the non-linear relationship between age and in-hospital mortality, age-restricted cubic spline adjusted logistic regression models were constructed for sex and the comorbidities [17].

R mediation package 4.5.0 was used to perform the mediation analysis [18]. The mediation analysis tests whether a clinical variable (in this case age 70+) affects outcome through a mediator variable (in this case the co-morbidities) and to which extent. This divides the total effect of the model into a direct effect, called the average direct effect (ADE), and an indirect effect, called the average causal mediation effect (ACME). The proportion of ADE and ACME and the 95% confidence intervals (using 1000x bootstrapping) were calculated and visualized for each comorbidity studied. Statistical analyses were conducted in R, and figures were made using the packages ggplot2 and forest plot [19–21]. Statistical significance was defined as a *P* value < 0.05.

## Results

### Patient characteristics

In total, 4806 patients fulfilled the inclusion criteria and were included in this study. Clinical characteristics stratified by age < 70 and ≥ 70 years are outlined in Table 1. The median age was 69 [58–77] years, and approximately two-third were men (63%, *N* = 3051). All pre-existing comorbidities were more prevalent in the elderly (aged ≥70 years, all *p* < 0.001). Male patients more often had dyslipidemia (46% vs 39%), a history of cardiac disease (39% vs 30%, especially coronary artery disease and arrhythmias) and presence of two or more comorbidities when compared to women (39% vs 33%, Additional file 2).

**Table 1:** Clinical characteristics of patients aged < 70 and aged ≥70 (70+) years

	< 70 (*N* = 2504)	70+ (*N* = 2302)	Total (*N* = 4806)	*P*-value
Clinical Presentation				
Age, years	58 [51–64]	77 [73–82]	69 [58–77]	< 0.001
Female	910 (36%)	845 (37%)	1755 (37%)	0.793
BMI, kg/m^2^	27.9 [25.1–31.2]	26.7 [24.0–30.1]	27.3 [24.5–30.8]	< 0.001
Temperature, °C	37.9 ± 1.1	37.7 ± 1.1	37.8 ± 1.1	< 0.001
Heart rate, bpm	91 [80–103]	86 [75–99]	89 [77–101]	< 0.001
Systolic BP, mmHg	133 ± 21	136 ± 24	135 ± 23	< 0.001
Diastolic BP, mmHg	78 ± 14	75 ± 15	77 ± 15	< 0.001
Breathing rate, rpm	22 [18–26]	21 [18–26]	22 [18–26]	0.620
Oxygen saturation, So2%	95 [92–97]	95 [92–96]	95 [92–97]	< 0.001
Medical History, n (%)				
Hypertension	1036 (41%)	1720 (75%)	2756 (57%)	< 0.001
Diabetes Mellitus	512 (20%)	701 (31%)	1213 (25%)	< 0.001
Dyslipidemia	764 (31%)	1303 (57%)	2067 (43%)	< 0.001
Chronic Kidney Disease	127 (5%)	392 (17%)	519 (11%)	< 0.001
COPD	206 (8%)	373 (16%)	579 (12%)	< 0.001
Cardiac disease	488 (20%)	1218 (53%)	1706 (36%)	< 0.001
Arrhyth./Conduc.	160 (6%)	612 (27%)	772 (16%)	< 0.001
Heart Failure	46 (2%)	228 (10%)	274 (6%)	< 0.001
Coronary Artery Disease	249 (10%)	574 (25%)	823 (17%)	< 0.001
Valvular Heart Disease	50 (2%)	201 (9%)	251 (5%)	< 0.001
Comorbidity count				< 0.001
0 comorbidities	1073 (43%)	250 (11%)	1323 (28%)	
1–2 comorbidities	896 (36%)	842 (37%)	1738 (36%)	
> 2 comorbidities	535 (21%)	1210 (53%)	1745 (36%)	

*Arrhyth* arrhythmias, *BMI* body mass index, *BP* blood pressure, *Conduc* conduction disorders, *COPD* chronic obstructive pulmonary disease

**Fig. 1:**
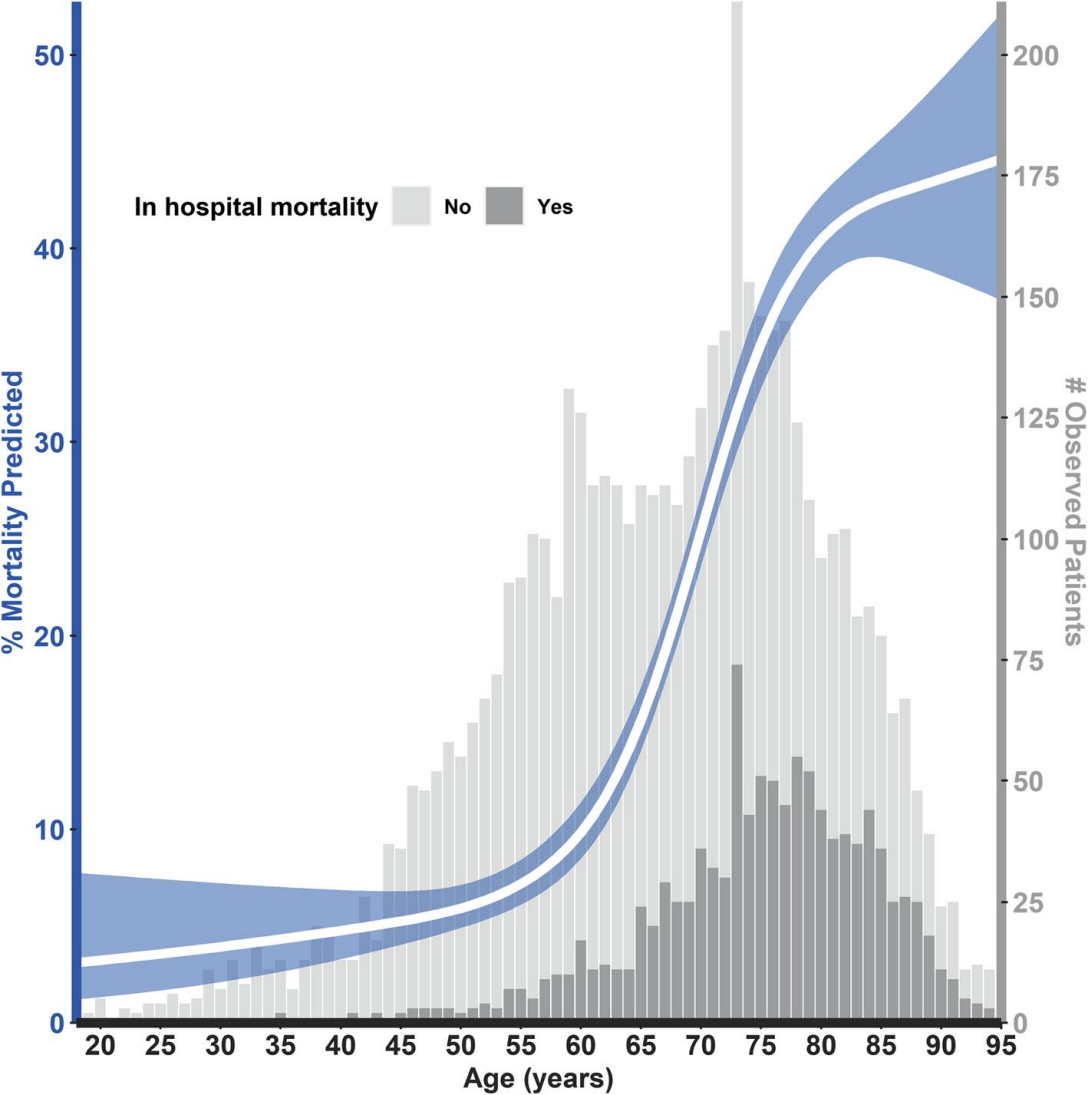
Predicted and observed in-hospital mortality (univariable age-restricted cubic spline adjusted). **% Mortality predicted**: The white line represents the predicted mortality rates at different ages, accompanied by 95% confidence intervals in blue. **# Observed patients:** The grey bars represent the number of patients observed within the different age categories. The light grey bars are the patients that were discharged without palliative care. The dark grey bars are the patients in which in-hospital mortality (or palliative discharge) occurred

Median duration of hospitalization was 7 [4 - 15] days. In-hospital mortality (*N *  = 1066, 96%) or palliative discharge (*N* = 42, 4%) occurred in 1108 (23%) patients, of which 836 (76%) were aged 70+ and 272 (24%) were aged < 70 (*p* <  0.001; Table 2). The observed and predicted association between age (splined adjusted) and in-hospital mortality are shown in Fig. 1. Male sex and the individual comorbidities were more prevalent, and comorbidity count per patient was higher in the in-hospital mortality group (*p* < 0.001, Table 2). In total, 1312 (27%) patients were referred to the intensive care unit (ICU) during hospitalization, of which 446 were aged 70+ (34%) and 866 (66%) were aged < 70 (*p* < 0.001).

**Table 2:** Clinical characteristics of patients with and without event

	No mortality or Palliative Care (*N* = 3698)	Mortality or Palliative Care (*N* = 1108)	Total (*N* = 4806)	*P*-value
Clinical Presentation				
Age, years	65 [56–74]	76 [70–82]	69 [58–77]	< 0.001
Female	1423 (39%)	332 (30%)	1755 (37%)	< 0.001
BMI, kgm^−2^	27.5 [24.7–30.8]	26.8 [24.1–30.5]	27.3 [24.5–30.8]	0.010
Temperature, °C	37.8 ± 1.1	37.8 ± 1.1	37.8 ± 1.1	0.454
Heart rate, bpm	89 [78–100]	90 [77–103]	89 [77–101]	0.087
Systolic BP, mmHg	135 ± 22	134 ± 25	135 ± 23	0.301
Diastolic BP, mmHg	77 ± 14	73 ± 16	77 ± 15	< 0.001
Breathing rate, rpm	20 [17–25]	24 [19–28]	22 [18–26]	< 0.001
Oxygen saturation, So2%	95 [93–97]	94 [91–96]	95 [92–97]	< 0.001
Medical History				
Hypertension	1979 (54%)	777 (70%)	2756 (57%)	< 0.001
Diabetes Mellitus	835 (23%)	378 (34%)	1213 (25%)	< 0.001
Dyslipidemia	1479 (40%)	588 (53%)	2067 (43%)	< 0.001
Chronic Kidney Disease	311 (8%)	208 (19%)	519 (11%)	< 0.001
COPD	389 (11%)	190 (17%)	579 (12%)	< 0.001
Cardiac disease	1170 (32%)	536 (48%)	1706 (36%)	< 0.001
Arrhyth./Conduc.	509 (14%)	263 (24%)	772 (16%)	< 0.001
Heart Failure	168 (5%)	106 (10%)	274 (6%)	< 0.001
Coronary Artery Disease	540 (15%)	283 (26%)	823 (17%)	< 0.001
Valvular Heart Disease	155 (4%)	96 (9%)	251 (5%)	< 0.001
Comorbidity count				< 0.001
0 comorbidities	1164 (32%)	159 (14%)	1323 (28%)	
1–2 comorbidities	1354 (37%)	384 (35%)	1738 (36%)	
> 2 comorbidities	1180 (32%)	565 (51%)	1745 (36%)	

*Arrhyth* arrhythmias, *BMI* body mass index, *BP* blood pressure, *Conduc* conduction disorders, *COPD* chronic obstructive pulmonary disease

### Univariable and multivariable associations and mediation effect of comorbidities and in-hospital mortality

**Fig. 2:**
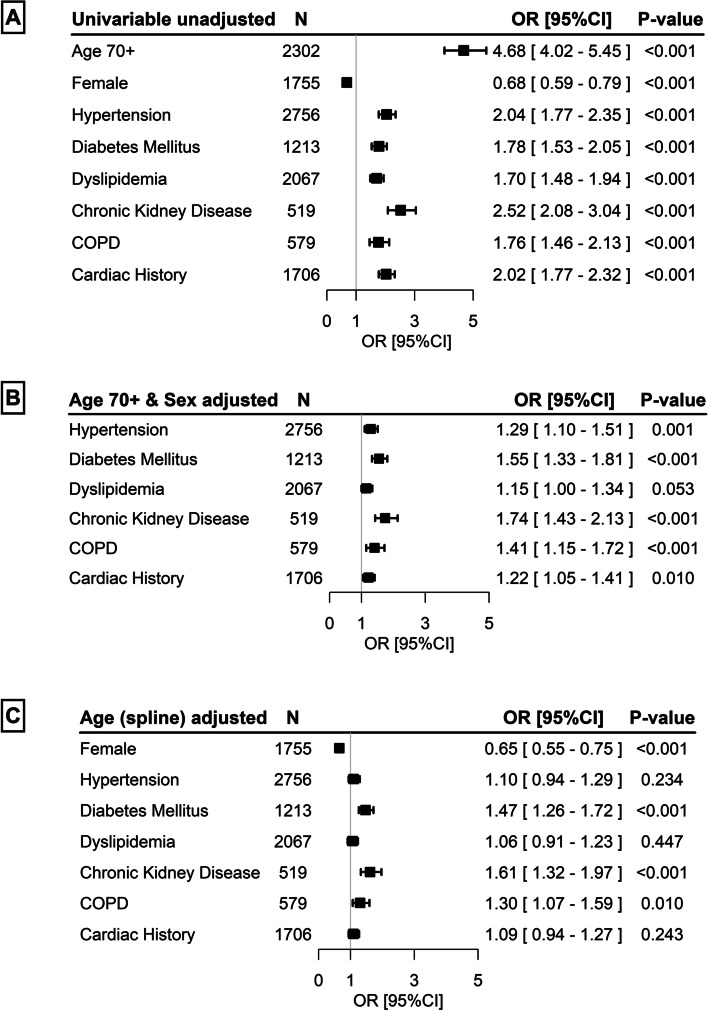
Associations of age and comorbidities with in-hospital mortality. **A)** Univariable association of age 70+, sex, and comorbidities with in-hospital mortality; **B)** Association of all comorbidities after adjustment for age 70+ and sex; **C)** Association of sex and all comorbidities after age spline adjustment. COPD = chronic obstructive pulmonary disease

Higher age (70+), male sex, and the individual comorbidities were all significantly associated with in-hospital mortality in univariable analysis (all *p* < 0.001; Fig. 2A). Hypertension, DM, CKD, COPD and a history of cardiac disease remained significantly associated after adjustment for age 70+ and sex (hypertension odds ratio [OR] 1.29, 95% confidence interval [CI] 1.10–1.51, *p* = 0.001; DM OR 1.55 [1.33–1.81], *p* < 0.001; dyslipidemia OR 1.15 [1.00–1.34], *p* = 0.053; CKD OR 1.74 [1.43–2.13], *p* < 0.001; COPD OR 1.41 [1.15–1.72], *p* < 0.001; history of cardiac disease OR 1.22 [1.05–1.41], *p* = 0.010, Fig. 2B). Additionally, given the non-linear relationship between age and in-hospital mortality, age-spline adjusted analysis was performed for the association of sex and all comorbidities with in-hospital mortality (Fig. 2C). The effects were attenuated after age-spline adjustment, only leaving sex, DM, CKD, and COPD significantly associated with in-hospital mortality (female OR 0.65 [0.55–0.75], *p* < 0.001; DM OR 1.47 [1.26–1.72], *p* < 0.001; CKD OR 1.61 [1.32–1.97], *p* < 0.001; COPD OR 1.30 [1.07–1.59], *p* = 0.010, Fig. 2C).

Uni- and multivariable (spline-adjusted) analysis of comorbidity count revealed a significant, univariable association with in-hospital mortality (*p* < 0.001; Additional file 3), which remained significant in the multivariable model with an attenuated effect. In the age-spline multivariable adjusted analysis, the association of the comorbidity count was not significant for a comorbidity count of 1–2 (OR 1.15 [0.9–1.4]) and attenuated for > 2 comorbidity count (OR 1.4 [1.1–1.7]). 

**Fig. 3:**
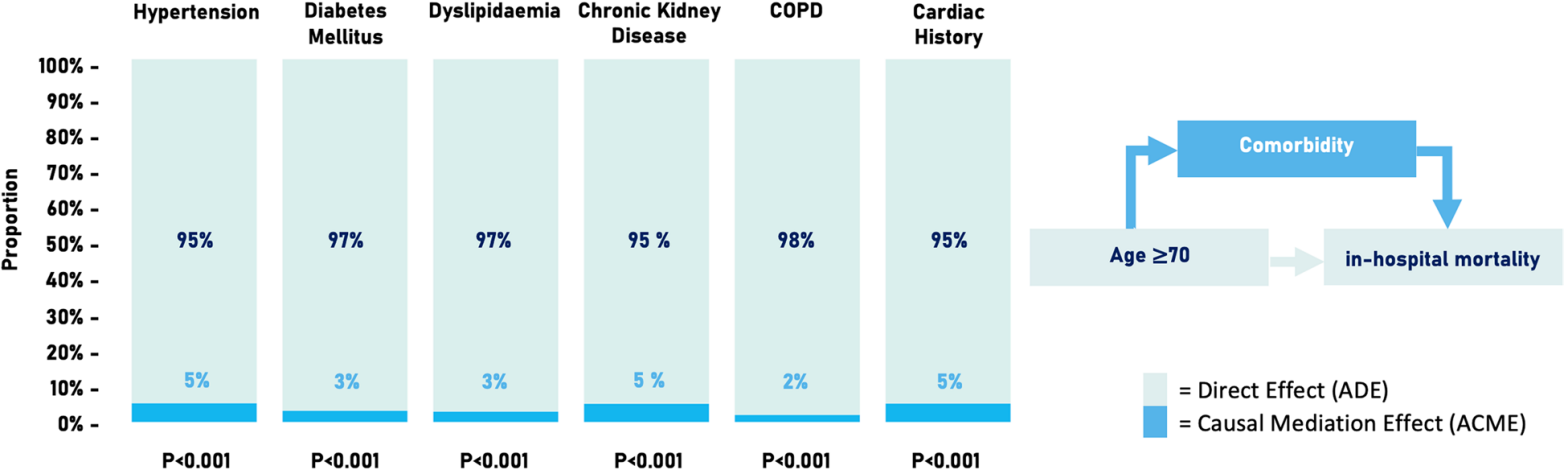
Mediation analysis with age ≥ 70 years as independent predictor, the individual comorbidities as mediator and in-hospital mortality as outcome. All comorbidities partly mediated the effect of age on in-hospital mortality (all *p*-values < 0.001). The mediation effect was 5% (95% CI 2–9%) for hypertension, 3% (95% CI 2–4%) for diabetes mellitus, 3% (95% CI 0.4–6%) for dyslipidemia, 5% (95% CI 3–7%) for chronic kidney disease, 2% (95% CI 1–3%) for COPD, and 5% (95% CI 2–9%) for history of cardiac disease. COPD = chronic obstructive pulmonary disease

All individual comorbidities significantly mediated the association between age 70+ and in-hospital mortality (ACME; Fig. 3). However, the proportion of this eff ect was below 6% for all comorbidities, and thereby the increased risk of in-hospital mortality in the elderly was explained mainly by the direct effect (ADE; Fig. 3) of age 70+ within this analysis (Fig. 3). Additionally, a mediation analysis was performed to quantify the mediation effect of a comorbidity count > 2 on the association between age and in-hospital mortality. Although the proportion of this effect was higher than the comorbidities separately, it remained minimal (8%, *p* < 0.001, Additional file 4).

Age-spline plots showed a steep increase in the predicted risk of in-hospital mortality from 60 years and higher (Fig. 1; analysis stratified by sex and the individual comorbidities is shown in Additional file 5). Correspondingly, in-hospital mortality rates were significantly lower in patients < 60 years when compared to the older patients (age 60+ years, 6% (*N* = 87) vs 30% (*N* = 1021), *p* < 0.001; Additional file 6). All comorbidities also occurred less frequently in this younger patient group (Additional file 6). None of the co-morbidities, except for history of cardiac disease, were significantly associated with in-hospital mortality in univariable analysis in patients < 60 years (hypertension OR 1.31 [0.83–2.06], *p* = 0.240; DM OR 1.38 [0.82–2.35], *p* = 0.228; dyslipidemia OR 1.26 [0.76–2.10], *p* = 0.367; CKD OR 2.33 [0.96–5.66], *p* = 0.062; COPD OR 0.62 [0.19–2.00], *p* = 0.421; history of cardiac disease OR 1.90 [1.10–3.27], *p* = 0.021, Additional file 7). In the total patient population, age 60+ was significantly associated with in-hospital mortality (OR 6.35[5.05–7.98], *p* < 0.001; Additional file 7A).

##  Discussion

This study reveals that higher age is the main determinant of COVID-19 related in-hospital mortality in the Netherlands. While pre-existing comorbidities (including hypertension, DM, dyslipidemia, CKD, COPD, and cardiac diseases) are more prevalent in the elderly, their mediation effect on COVID-19 related in-hospital mortality is minimal. 

Over the last months, studies revealed multiple risk factors independently associated with COVID-19 related (in-hospital) mortality, including higher age, pre-existing co-morbidities, and male sex, which is in line with current findings [9, 10, 22–25]. A recent study of 2273 COVID-19 hospitalized Dutch patients showed that a mortality prediction model using ten clinical features including age, number of home medications, admission blood values urea nitrogen/LDH/albumin, oxygen saturation, blood gas pH and history of chronic cardiac disease, improved discrimination over age-based decision rules only [26]. Nonetheless, no data regarding to which extent age and cardiovascular comorbidities contributed to the mortality risks were provided. We show that the contributing effect of pre-existing comorbidities is minimal relative to age.

In the Netherlands, the vast majority (90%) of deceased patients due to COVID-19 is over 70 years of age. The prevalence of comorbidities among hospitalized COVID-19 patients is particularly high, especially in the elderly [7, 8]. The current study included 4806 patients from 45 Dutch - both academic and local - hospitals, accounting for around 40% of all COVID-19 related hospitalizations between January 2020 and July 2020 in the Netherlands [7]. Therefore, this study population adequately represents the total hospitalized population during the first COVID-19 pandemic wave in the Netherlands. The higher prevalence of male COVID patients observed in the current registry and the increased mortality risk of males is in line with previous findings and likely due to a less effective viral control of the immune system in males [24, 27], together with the higher comorbidity rates in males compared to females [24, 28]. The observed association between a medical history of a cardiac disease and in-hospital mortality in the young has been studied and discussed before by current consortium, which is mainly driven by the presence of severe (NYHA≥3) heart failure [29].

It is important to notice that during the first wave of COVID-19, the median age of people referred to the ICU department decreased when the total number of patients referred to the ICU department increased [30]. Younger (< 70+) patients were possibly  more likely to be referred to the ICU department due to bed shortages. However, the percentage of patients aged 70+ in the COVID-19 related  mortality group in the current study (76%) is lower than the overall percentage of subjects aged 70+ of the COVID-19 related deaths in the Netherlands in 2020 (90%) [31]. This likely results from fewer referrals of elderly patients to the hospitals (possibly even independent of the presence of comorbidities) due to the overwhelming stress on hospitals during the first COVID-19 wave. Nonetheless, even in the group aged < 60 years - in whom likely no or limited referral “restrictions” were present - the effect of pre-existing comorbidities on mortality was limited in the current study, indicating that age as such is likely the main driver of COVID-19 related in-hospital mortality.

Our study revealed that pre-existing co-morbidities, highly prevalent in the elderly, contributed minimally to in-hospital mortality when compared to age. Moreover, the contribution of the comorbidities to outcome in patients aged < 60 years was limited to a history of cardiac disease without any significant association for DM, CKD, and COPD. As a result, the present study stresses the need for primary preventive efforts to protect the elderly (males) from an infection with SARS-CoV-19.

## Limitations

There are some study limitations that need to be addressed, including its retrospective design. Additionally, data concerning the patients’ frailty and the reasons for not referring patients to the hospital or the ICU, were not collected within this registry. To which extent the lower hospital and ICU referral rate of the elderly and frailty contributed to the in-hospital mortality rate could therefore not be assessed and requires further research. Such research could help to better understand the association of higher age and in-hospital mortality beyond the comorbidities and might guide decision making on treatment, counselling and admission to high care facilities in the future.

## Conclusion

Age is the main determinant of COVID-19 related in-hospital mortality, which is negligibly mediated by pre-existing comorbidities in the Netherlands.

## Supplementary Information

**Additional file 1.** Supplemental methods.

**Additional file 2.** Clinical characteristics of males and females.

**Additional file 3.** Univariable, and Multivariable and Age cubic Spline adjusted association of Age, Gender, and comorbidity count with in-hospital mortality.

**Additional file 4.** Mediation analysis with Age ≥ 70 as independent predictor, multi-comorbidity (> 2 comorbidities) as mediator and in-hospital mortality as outcome.

**Additional file 5. **Age-spline adjusted associations with predicted in-hospital mortality, stratified for: **A)** sex; **B)** number of comorbidities; **C)** hypertension; **D)** diabetes mellitus; **E)** dyslipidemia; **F)** chronic kidney disease; **G)** chronic obstructive pulmonary disease (COPD); **H)** cardiac disease.

**Additional file 6.** Clinical characteristics of patients below and ≥ 60 years of age.

**Additional file 7. A)** Univariable association of Age 60+ with in-hospital mortality in the total cohort **B)** Univariable association of sex and comorbidities with in-hospital mortality in patients younger than 60 years.

## Data Availability

The datasets used and/or analyzed during the current study are available from the corresponding author on reasonable request.

## Abbreviations

ADE: Average direct effect
ACME: Average causal mediation effect
CKD: Chronic kidney disease
COPD: Chronic obstructive pulmonary disease
SARS-CoV-19: severe acute respiratory syndrome coronavirus 2
DM: Diabetes mellitus
ICU: Intensive care unit
