# Supplementary material for: Age is the main determinant of COVID-19 related in-hospital mortality with minimal impact of pre-existing comorbidities, a retrospective cohort study
Source: BMC Geriatr. 2022 Mar 5;22:184. doi: 10.1186/s12877-021-02673-1 (PMC8897728; doi:10.1186/s12877-021-02673-1)
Supplement: Supplementary file 1 — Additional file 1. Supplemental methods. [file 12877_2021_2673_MOESM1_ESM.docx]

**Additional file 1.** Supplemental methods

Hypertension was defined as: a reported history of hypertension diagnosed and treated with medication, diet and/or exercise, or current pharmacological anti-hypertensive therapy. Diabetes Mellitus was defined as a reported history of diabetes mellitus diagnosed and/or treated by a physician. Dyslipidemia was defined as a reported history of dyslipidemia diagnosed and/or treated by a physician. Cut-off values for dyslipidemia were defined as: Total cholesterol greater than 200 mg/dl (5.18 mmol/l) Low-density lipoprotein (LDL) greater than or equal to 130 mg/dl (3.37 mmol/l) High-density lipoprotein (HDL) less than 40 mg/dl (1.04 mmol/l) in men and less than 50 mg/dl (1.30 mmol/l) in women. Chronic kidney disease (CKD) was defined as a reported history of CKD diagnosed and/or treated by a physician. Cut-off values for CKD were defined as: eGFR < 60 ml/min/1.73 m2 (for >3months) or urine albumin-creatinine ratio ≥ 3 mg/mmol. Chronic obstructive pulmonary disease (COPD) was present when a patient had a history of COPD diagnosed and/or treated by a physician. Cut-off values for COPD are defined as: FEV1 / FVC < 5th percentile of reference population (z-score < -1.64).
