# Supplementary material for: Age is the main determinant of COVID-19 related in-hospital mortality with minimal impact of pre-existing comorbidities, a retrospective cohort study
Source: BMC Geriatr. 2022 Mar 5;22:184. doi: 10.1186/s12877-021-02673-1 (PMC8897728; doi:10.1186/s12877-021-02673-1)
Supplement: Supplementary file 2 — Additional file 2. Clinical characteristics of males and females. [file 12877_2021_2673_MOESM2_ESM.docx]

**Additional file 2.** Clinical characteristics of males and females.

|  | Males  (N=3051) | Females  (N=1755) | P-value |
| --- | --- | --- | --- |
| Clinical Presentation |  |  |  |
| Age, years | 69 [58-76] | 69 [57-78] | 0.984 |
| BMI, kgm^-2^ | 27.1 [24.6-30.0] | 28.1 [24.4-32.2] | < 0.001 |
| Temperature, °C | 37.8 ±1.1 | 37.7 ±1.1 | 0.030 |
| Heart rate, bpm | 88 [77-101] | 90 [79-100] | 0.138 |
| systolic BP, mmHg | 135 ±22 | 134 ±23 | 0.097 |
| diastolic BP, mmHg | 77 ±14 | 79 ±15 | 0.020 |
| Breathing rate, rpm | 22 [18-26] | 20 [17-25] | <0.001 |
| Oxygen saturation, So2% | 95 [92-97] | 95 [93-97] | <0.001 |
| Medical History |  |  |  |
| Hypertension | 1741 (57%) | 1015 (58%) | 0.603 |
| Diabetes Mellitus | 762 (25%) | 451 (26%) | 0.579 |
| Dyslipidemia | 1389 (46%) | 678 (39%) | <0.001 |
| Chronic Kidney Disease | 340 (11%) | 179 (10%) | 0.310 |
| COPD | 357 (12%) | 222 (12%) | 0.331 |
| Cardiac disease | 1178 (39%) | 528 (30%) | <0.001 |
| Arrhyth./Conduc. | 533 (18%) | 239 (14%) | <0.001 |
| Heart Failure | 170 (6%) | 104 (6%) | 0.610 |
| Coronary Artery Disease | 639 (21%) | 184 (11%) | <0.001 |
| Valvular Heart Disease | 159 (5%) | 92 (5%) | 0.963 |
| Comorbidity count |  |  | <0.001 |
| 0 comorbidities | 842 (28%) | 481 (27%) |  |
| 1-2 comorbidities | 1035 (34%) | 703 (40%) |  |
| >2 comorbidities | 1174 (39%) | 571 (33%) |  |
| Outcome |  |  |  |
| Mortality/palliative care | 776 (25%) | 332 (19%) | <0.001 |

Arrhyth = arrhythmias; BMI = body mass index; BP = blood pressure; Conduc = conduction disorders; COPD = chronic obstructive pulmonary disease.
