# Supplementary material for: Age is the main determinant of COVID-19 related in-hospital mortality with minimal impact of pre-existing comorbidities, a retrospective cohort study
Source: BMC Geriatr. 2022 Mar 5;22:184. doi: 10.1186/s12877-021-02673-1 (PMC8897728; doi:10.1186/s12877-021-02673-1)

**Additional file 3.** Univariable, and Multivariable and Age cubic Spline adjusted association of Age, Gender, and comorbidity count with in-hospital mortality.


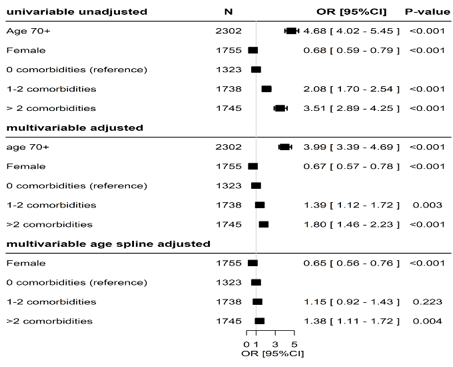

Supplement: Supplementary file 3 — Additional file 3. Univariable, and Multivariable and Age cubic Spline adjusted association of Age, Gender, and comorbidity count with in-hospital mortality. [file 12877_2021_2673_MOESM3_ESM.docx]
