# Supplementary material for: Age is the main determinant of COVID-19 related in-hospital mortality with minimal impact of pre-existing comorbidities, a retrospective cohort study
Source: BMC Geriatr. 2022 Mar 5;22:184. doi: 10.1186/s12877-021-02673-1 (PMC8897728; doi:10.1186/s12877-021-02673-1)
Supplement: Supplementary file 4 — Additional file 4. Mediation analysis with Age ≥ 70 as independent predictor, multi-comorbidity (> 2 comorbidities) as mediator and in-hospital mortality as outcome. [file 12877_2021_2673_MOESM4_ESM.docx]

**Additional file 4.** Mediation analysis with Age ≥70 as independent predictor, multi-comorbidity (>2 comorbidities) as mediator and in-hospital mortality as outcome.


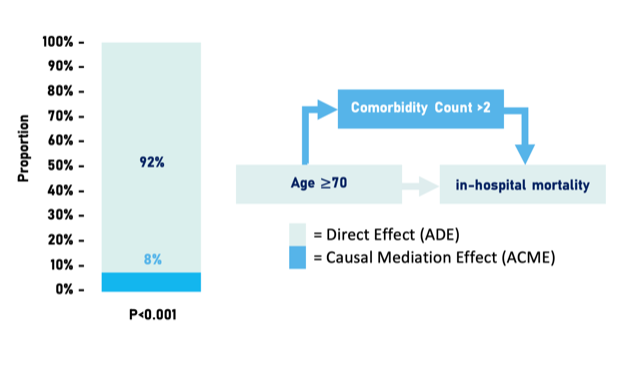


The comorbidity count >2 partly mediated the effect of age on in-hospital mortality (p <0.001). The mediation effect was 8% (95% CI 4-11%).
