# Supplementary material for: Age is the main determinant of COVID-19 related in-hospital mortality with minimal impact of pre-existing comorbidities, a retrospective cohort study
Source: BMC Geriatr. 2022 Mar 5;22:184. doi: 10.1186/s12877-021-02673-1 (PMC8897728; doi:10.1186/s12877-021-02673-1)
Supplement: Supplementary file 5 — Additional file 5. Age-spline adjusted associations with predicted in-hospital mortality, stratified for: A) sex; B) number of comorbidities; C) hypertension; D) diabetes mellitus; E) dyslipidemia; F) chronic kidney disease; G) chronic obstructive pulmonary disease (COPD); H) cardiac disease. [file 12877_2021_2673_MOESM5_ESM.docx]

**Additional file 5.** Age-spline adjusted associations with predicted in-hospital mortality, stratified for: **A)** sex; **B)** number of comorbidities; **C)** hypertension; **D)** diabetes mellitus; **E)** dyslipidemia; **F)** chronic kidney disease; **G)** chronic obstructive pulmonary disease (COPD); *
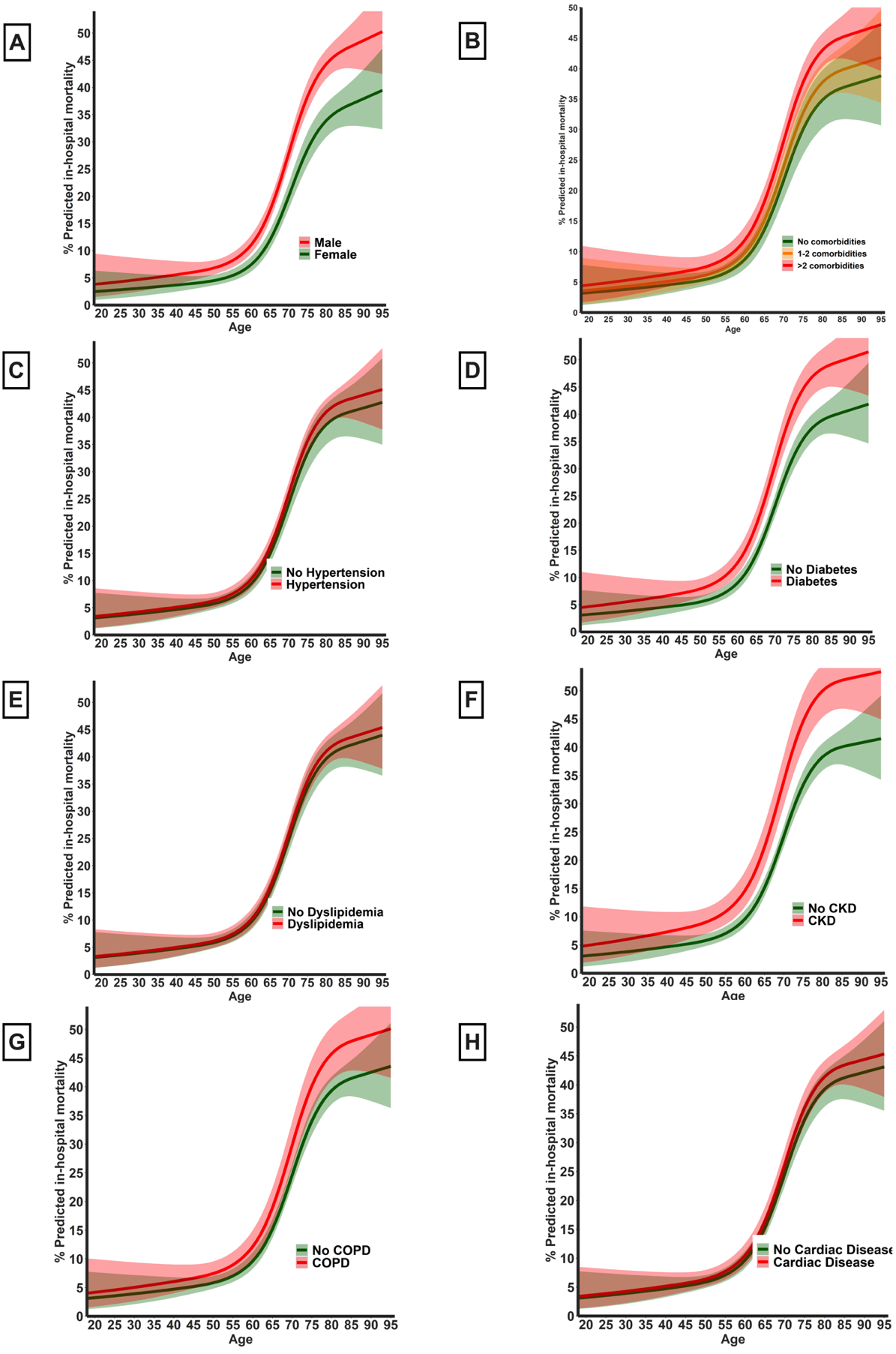
***H)** cardiac disease.
