# Supplementary material for: Age is the main determinant of COVID-19 related in-hospital mortality with minimal impact of pre-existing comorbidities, a retrospective cohort study
Source: BMC Geriatr. 2022 Mar 5;22:184. doi: 10.1186/s12877-021-02673-1 (PMC8897728; doi:10.1186/s12877-021-02673-1)
Supplement: Supplementary file 6 — Additional file 6. Clinical characteristics of patients below and ≥ 60 years of age. [file 12877_2021_2673_MOESM6_ESM.docx]

**Additional file 6.** Clinical characteristics of patients below and ≥60 years of age.

|  | <60  (N=1385) | 60+  (N=3421) | P-value |
| --- | --- | --- | --- |
| Clinical Presentation |  |  |  |
| Age, years | 52 [46-56] | 73 [67-80] | <0.001 |
| Female | 532 (38%) | 1223 (36%) | 0.083 |
| BMI, kg/m^2^ | 28.5 [25.2-31.9) | 26.9 [24.3-30.4] | <0.001 |
| Temperature, °C | 37.9 ±1.1 | 37.7 ±1.1 | <0.001 |
| Heart rate, bpm | 93 [82-105] | 87 [76-100] | <0.001 |
| Systolic BP, mmHg | 132 ±20 | 135 ±23 | <0.001 |
| Diastolic BP, mmHg | 80 ±14 | 75 ±15 | <0.001 |
| Breathing rate, rpm | 22 [18-27] | 21 [18-26] | 0.012 |
| Oxygen saturation, So2% | 95 [93-97] | 95 [92-96] | <0.001 |
| Medical History, n(%) |  |  |  |
| Hypertension | 431 (31%) | 2325 (68%) | <0.001 |
| Diabetes Mellitus | 237 (17%) | 976 (29%) | <0.001 |
| Dyslipidemia | 282 (20%) | 1785 (52%) | <0.001 |
| Chronic Kidney Disease | 46 (3%) | 473 (14%) | <0.001 |
| COPD | 74 (5%) | 505 (15%) | <0.001 |
| Cardiac disease | 175 (13%) | 1531 (45%) | <0.001 |
| Arrhyth./Conduc. | 49 (4%) | 723 (21%) | <0.001 |
| Heart Failure | 16 (1%) | 258 (8%) | <0.001 |
| Coronary Artery Disease | 75 (5%) | 748 (22%) | <0.001 |
| Valvular Heart Disease | 12 (1%) | 239 (7%) | <0.001 |
| Comorbidity count |  |  | <0.001 |
| 0 comorbidities | 757 (55%) | 566 (17%) |  |
| 1-2 comorbidities | 444 (32%) | 1294 (38%) |  |
| >2 comorbidities | 184 (13%) | 1561 (46%) |  |
| Outcome |  |  |  |
| Mortality/palliative care | 87 (6%) | 1021 (30%) | < 0.001 |

Arrhyth = arrhythmias; BMI = body mass index; BP = blood pressure; Conduc = conduction disorders; COPD = chronic obstructive pulmonary disease.
