# Supplementary material for: Age is the main determinant of COVID-19 related in-hospital mortality with minimal impact of pre-existing comorbidities, a retrospective cohort study
Source: BMC Geriatr. 2022 Mar 5;22:184. doi: 10.1186/s12877-021-02673-1 (PMC8897728; doi:10.1186/s12877-021-02673-1)

**Additional file 7. A)** Univariable association of Age 60+ with in-hospital mortality in the total cohort **B)** Univariable association of sex and comorbidities with in-hospital mortality in patients younger than 60 years.

**A**

**B**


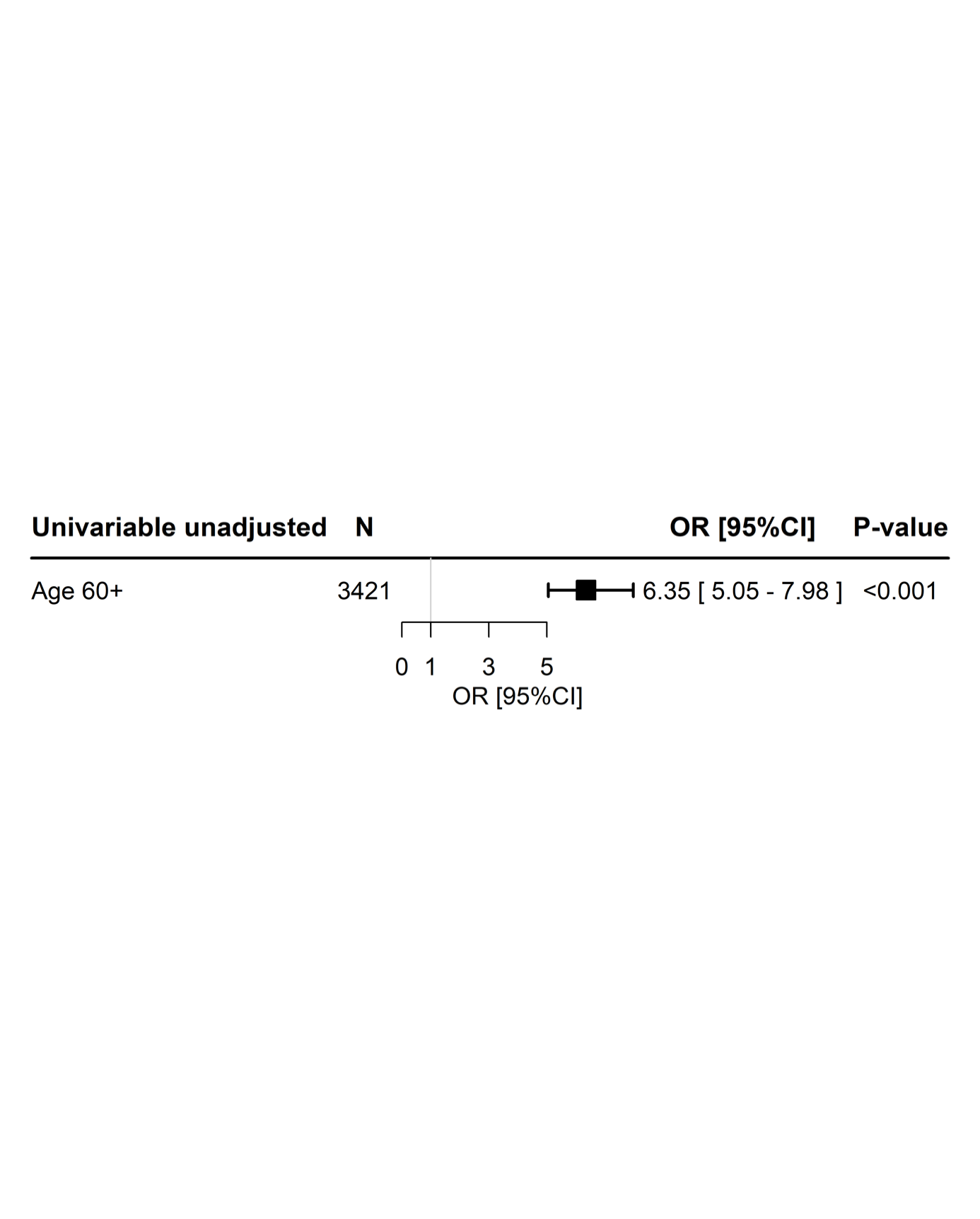

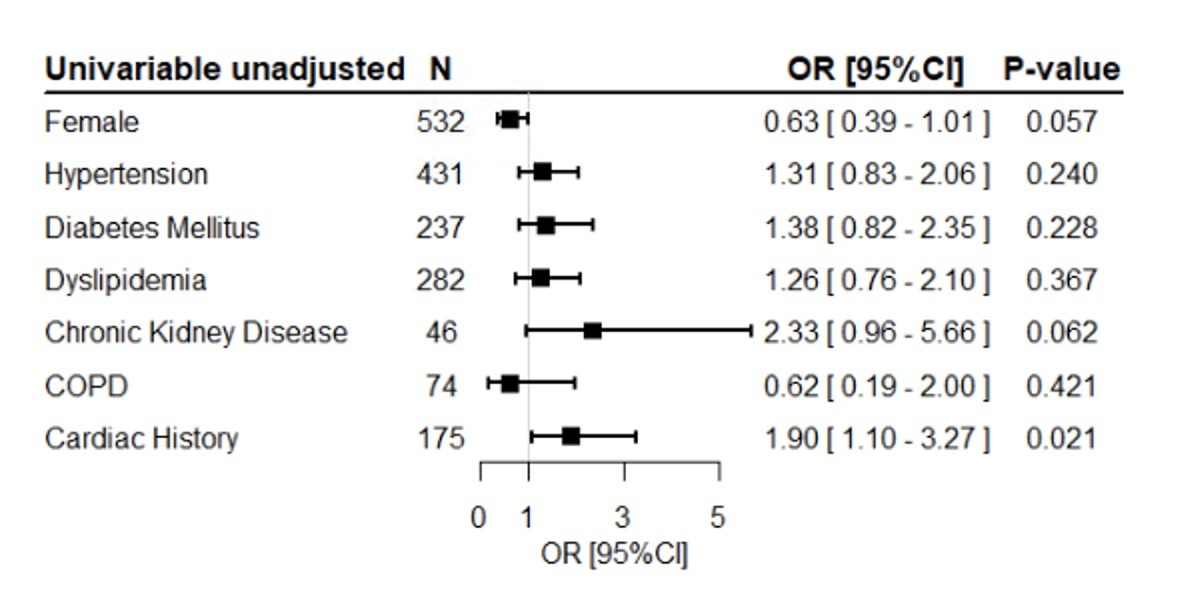

Supplement: Supplementary file 7 — Additional file 7. A) Univariable association of Age 60+ with in-hospital mortality in the total cohort B) Univariable association of sex and comorbidities with in-hospital mortality in patients younger than 60 years. [file 12877_2021_2673_MOESM7_ESM.docx]
